# A descriptive study of self-medication practices among Sri Lankan national level athletes

**DOI:** 10.1186/s13104-017-2579-8

**Published:** 2017-07-06

**Authors:** A. D. A. Fernando, L. M. H. Bandara, H. M. S. T. Bandara, S. Pilapitiya, A. de Silva

**Affiliations:** 1 Department of Physiology, Faculty of Medicine, University of Colombo, No 25, Kynsey Road, Colombo 8, Sri Lanka; 2 Sri Lanka Anti-doping Agency, Institute of Sports Medicine, Independence Avenue, Colombo 7, Sri Lanka

**Keywords:** Self-medication, Athletes, Allopathic medicine, Herbal/traditional medicine, Sri Lanka

## Abstract

**Background:**

Intake of medicines and supplements is widespread among the professional athletes in developed countries and there are reports to suggest inappropriate self-administration of medicine. Data from South Asia on this area is lacking. This study examined self-medication practices with regard to use of allopathic and herbal/traditional medicines among national -level Sri Lankan athletes.

**Results:**

209 athletes from 15 national sport teams were assessed using an anonymous, interviewer administered questionnaire. Self-medication practices during the 3 months before data collection were evaluated. 60.8% athletes practiced self-medication. 58.3 and 9.4% consumed western and herbal/traditional medicines respectively, while a third used both. The most common symptom for which self-medication was practiced was musculoskeletal pain (73.2%). Oral non-steroidal anti-inflammatory drugs (NSAIDs) and antibiotics were used by 15.7 and 7.1% respectively. Musculoskeletal pain was the predominant symptom that prompted the use of allopathic medicines, while the majority of athletes with upper respiratory tract symptoms being the predominant symptoms, consumed herbal/traditional medicines. Two different commercially available preparations of herbal mixtures were consumed by 15.7 and 15%. Pain prophylaxis during or prior to a sport event was reported by 20.1%, mainly with topical medicines. Medicines were obtained by direct request from a pharmacy without an authorized prescription by a majority (77.2%), followed by using an old prescription in 12.6%.

**Conclusions:**

This study finds that self-medication with both allopathic and herbal/traditional preparations among athletes in a Sri Lanka is high. The use of oral NSAIDs without an authorized prescription in a significant number of athletes is a potential health risk. Frequency of oral NSAID use is lower than that is reported in non-Asian studies from developed countries. The use of herbal/traditional medications increases the likelihood of inadvertent doping. Enhancing awareness regarding risk of such practices among athletes, trainers, pharmacists and prescribers is essential.

## Background

Self-medication is the selection and use of medicines, including herbal and traditional products, by individuals to treat self-recognised illnesses or symptoms [1]. Self-medication allows the patient/consumer to take an active role in his or her own care. However, it is not a completely safe health behaviour, particularly in the case of unreliable or irresponsible practice [2]. Potential risks of self-medication include incorrect self-diagnosis, incorrect dosage, incorrect choice of therapy, masking of severe disease, drug interactions, adverse reactions, dependence and abuse [2]. A high intake of medicines and nutritional supplements among professional athletes is widespread and is well documented in developed countries [3–6]. In most instances in developed countries, the medicines are prescribed by the team physicians. However, there is consistent data reporting self-administration of medicines that has raised concerns [4, 5, 7, 12]. The athletes use a range of medications to treat injuries, cure illnesses and obtain a competitive advantage [3]. Documented evidence is available on athletes using non-steroidal anti inflammatory drugs (NSAIDs), often in high doses and in combination, more often than age-matched controls, especially those competing in speed and power sports, soon before competition [3]. Similar findings are described for oral antibiotics as well [3]. Athletes have relatively unrestricted access to NSAIDs, as they are readily available over-the-counter preparations [10] in developed countries and are not prohibited as performance-enhancing drugs by the World Anti-Doping Agency (WADA) [7, 8], leading to frequent use of NSAIDs in treatment as well as pain prophylaxis prior to sporting events [8, 10]. In addition to the well-known adverse effects of NSAIDS, their prophylactic use masks pain and leads to muscle damage, causing a paradoxical situation compromising the musculoskeletal system [7–10]. The documented problems of self- medication in athletes is not only limited to untoward adverse drug reactions. It has been shown that the athletes who use analgesics prior to their events could be more prone to using doping substances [11].

A high prevalence of medicine use has been reported during many international sporting events such as FIFA (The Fédération Internationale de Football Association) World Cups in 2002 and 2006 [4] Athens Paralympic Games in 2004 [12] and Atlanta and Sydney Olympics [13]. A high intake of NSAIDs was common in all above, which was a cause for concern. Also, the use of inhaled β2 agonists was higher than expected in Atlanta and Sydney Olympics [13]. Triathletes in Brazil reported a high prevalence of NSAID use, limited awareness of the adverse effects and a high rate of non-prescribed use [6]. There is concern that self-medication with analgesics is prevalent among school athletes as well [14–16].

Considering the South Asian situation, self-medication and non-doctor prescribing of drugs among the general population have been reported from Nepal [17]. A Sri Lankan study on dietary supplement intake by national-level athletes reported widespread intake, a fewer number of supplements and limited awareness on risks and benefits of the supplements they were taking [18]. However, published data on self-administration of medicines among professional athletes in developing countries is sparse. The possibility of athletes practising self-medication is high among local athletes because of easy access and availability of medicines due to non-adherence to regulations on sale. An added phenomenon in Sri Lanka according to unpublished observations, is the use of herbal/traditional products as self-medication, a popular practice. Over the counter herbal medicinal products are used in many other parts of the world and their safety is questionable at times [19]. National surveys on the prevalence of complementary and alternative medicines are not available for most South Asian countries. Anecdotal evidence support popular use of over the counter preparations of herbal/traditional medications by local athletes. However, the actual use of such substances has not been studied, and often, the ingredients in such preparations have not been evaluated. Intake of herbal/traditional preparations without advice could cause adverse health effects and inadvertent doping issues for international level athletes.

It will be a useful first step to know the current prevalence, practices, and patterns and perceptions of athletes about self- medication before remedial measures can be taken to educate athletes, their trainers, and pharmacists on potential detrimental effects of using medication unsupervised and without proper awareness. This study evaluates some aspects of self-medication among Sri Lankan national-level athletes competing in different sporting events.

## Methods

The eligible study population consisted of members of the Sri Lankan national squads for volleyball, netball, track and field, wrestling, football, hockey, karate, wushu, archery, weight-lifting, badminton, gymnastics, water polo, Kabadi (a wrestling sport played in South Asia), cricket, rugby, basketball, swimming and table-tennis. All athletes aged >16 years and not participating in on-going tournaments during the period of data collection were invited to participate. The data collection was carried out in collaboration with the Institute of Sports Medicine, Ministry of Sports, Sri Lanka, at the institute premises, during April–July 2013.

An anonymized, interviewer administered questionnaire was used to obtain data and was pre-tested on 10 Colombo University athletes. The questionnaire was structured, taking into consideration, the patterns of self-medication/supplementation use among athletes in previous published work [5, 17, 18] and unpublished observations on accessibility and availability of medicines in the local market and patterns of medicinal use in the local community. The questionnaire consisted of four domains that obtained data on age, sex, level of education, and number of years of performance, practice of self-medication for prophylaxis and symptomatic relief during the past 3 months, knowledge on aspects of self-medication and perceptions on self-medication. Symptoms for which relief was sought were classified as musculoskeletal pain, respiratory, gastro-intestinal, general and menstrual symptoms. Medications were classified as analgesics, antibiotics, respiratory medicines, anti-pyretics, herbals/traditional medicines and a final group as ‘other medications’ where the formulation was nonspecific.

Data were entered and analyzed using SPSS.v.20.0. Percentages and frequencies were used to describe data. Cross tabulations and Pearson’s Chi square tests were applied to assess significant differences between categorical variables. The level of significance was set at p ≤ 0.05. The primary outcome measures were the prevalence of self-medication practices, common symptoms for which self-medication was used and the variety of medications used by athletes. These outcome measures provided an indirect means of assessing patterns of self-medication usage and influencing factors.

Ethical clearance was obtained from the Ethics Review committee, Faculty of Medicine, University of Colombo, Sri Lanka. Verbal informed consent was obtained from all athletes after they were informed of the purpose of the questionnaire, use of obtained data to generate awareness regarding beneficial and adverse effects of self-medication and assurances of confidentiality and anonymity of data.

## Results

### Participants

209 national level athletes over the age of 16 years responded. A limited sociodemographic profile of the participants is shown in Table 1. Most athletes were male and 82.8% were <30 years of age. 139 (66.5%) had completed secondary education. Two-thirds of participants were employed (73.7%). The athletes included teams from Volleyball (n = 22), Netball (n = 15), Track and field (n = 24), Wrestling (n = 11), Football (n = 12), Hockey (n = 26), Karate (n = 20), Wushu (n = 10), Archery (n = 19), Weight-lifting (n = 7), Badminton (n = 5), Gymnastics (n = 4), Water polo (n = 21), Kabadi (n = 9) and Table-tennis (n = 4). In badminton, gymnastics and table tennis teams, the response rates were 42, 40 and 40% respectively. In all other teams, response rates were 100%. One hundred and thirty (62.2%) participants had already participated in international sporting events/competitions.

**Table 1:** Some selected sociodemographic characteristics of the athletes

Characteristic	
Age range	16–40 years
Gender (*n*)	
Male	122
Female	87
Education (*n*)	
Primary education	45
Secondary education	142
Tertiary education	22
Monthly income in USD (*n*) (1USD = 142 LKR)	
35–100	3
101–175	22
176–210	18
211–280	80
>280	57
Not declared	29

### Prevalence and frequency of practice of SM

127 (60.8%) participants reported self-medication with therapeutic intention during the past 3 months. 74 (58.3%) self-medicating athletes used allopathic medicines, 12 (9.4%) used herbal/traditional medicines, and 41 (32.3%), both types. 73 athletes (57.5%) used multiple medicines. Intake of 33 different medicines were reported (0.26 per athlete), of which 20 were allopathic (0.15 per athlete) and 13, herbal/traditional (0.10 per athlete). No differences in self-medication practices were seen across sex, age group, education level or monthly income. Frequency of self-medication differed across different sports teams with the kabadi and badminton teams having the highest frequency (100%), whereas the wrestling team had the lowest (27.3%) (p < 0.05).

### Indications for self-medication and medicinal groups

Out of 127 who had practiced self-medication, forty-two (33%) used pain prophylaxis prior or during a sport event. Modes of pain prophylaxis administration were as follows; spray (n = 35, 83.3%), gel (n = 14, 33.3%), oral (n = 9, 21.4%) and adhesive pain relief patch (n = 1, 2.4%). None took herbal/traditional medicines for pain prophylaxis.

Indications for self-medication for symptom relief are given in Table 2.

**Table 2:** Indications for self-medication

Symptom	Frequency (n = 127)	Proportion of all athletes (n = 209)
Pain	93 (73.2%)	44.5%
Respiratory symptoms		
Upper respiratory tract symptoms	40 (31.5%)	19.1%
Lower respiratory tract symptoms	30 (23.6%)	14.6%
Fever	24 (18.9%)	11.4%
Dysmenorrhoea	13 (10.2%)	6.2%
Muscle cramps	10 (7.9%)	4.8%
Abdominal pain/colics	3 (2.4%)	1.4%
Superficial wound/skin rash	3 (2.4%)	1.4%
Other	5 (3.9%)	2.4

Musculoskeletal pain was the predominant symptom that prompted the use of allopathic medicines (p < 0.05) while the majority of participants with respiratory tract symptoms with cough (p < 0.05) and common cold (p < 0.05) being the predominant symptoms, consumed herbal/traditional medicines.

The groups of medications used by athletes are presented in the Table 3.

**Table 3:** Groups of medications and the proportion of athletes that used them

Medicinal group	Proportion
Allopathic medicines	
Paracetamol	82 (64.6%)
Other NSAIDs	20 (15.7%)
Antibiotics	09 (7.1%)
Antihistamines	09 (7.1%)
Vitamins	15 (11.8%)
Herbal/traditional medicines	
Commercially prepared mixture of herbals	20 (15.7%)
Paspanguwa (mixture of 5 herbs)	19 (15.0%)
False calumba (*Coscinium fenestratum*)	8 (6.3%)
Coriander (*Coriandrum sativum*)	5 (3.9%)
Other	7 (5.5%)

### Ways of obtaining medicines

The medications were obtained by direct request from a pharmacy without an authorized prescription by a majority (77.2%), followed by using an old prescription in 12.6%. 10.2% of athletes consumed medications that were acquired by their family members and 7.9% used medicines that they acquired from non-medical persons such as friends.

### Perceptions and awareness

The reasons for practicing self-medication was an assumption that the symptoms were not serious enough to warrant a visit to the doctor (31.0%), unavailability of a prescriber when needed (4.7%), busy schedule/travelling issues (6.3%) and lack of effectiveness of previously prescribed medicines (0.8%). No participant documented financial problems as a reason for self-medication.

One hundred and fifteen (90.6%) participants were aware of names (either generic or brand names) of the medicines they consumed and 3.2% reported upper gastrointestinal adverse effects after consuming medicines. Approximately half of the participants (53.5%) were aware that self-medication could be harmful for their health and performance. Awareness was similar across age groups, sex, education level or type of sport.

Over a third of athletes (37.0%) reported that their trainers were aware of self-medication practices. Family members promoted self-medication in 20 (15.7%) participants, followed by trainers (6.3%), doctors (3.1%), friends/colleagues (1.6%), physiotherapists (n = 2, 1.6%) and mass media (n = 2, 1.6%). A doctor influenced 31% of athletes on using pain prophylaxis with topical medicines, followed by trainers/physiotherapists (21.4%) and peers (11.9%).

One hundred and twenty (94.5%) persons who took medications perceived improved symptoms and 38 (29.9%) perceived improved performance in sport.

## Discussion

The highly competitive nature of modern-day sports and the economic impact of athletic performance at elite levels have driven athletes to play with injury and sickness [20], which has led to the widespread use of a variety medicines including analgesic agents in developed countries. Most studies in the developed world on intake of non-performance enhancing medicines have focussed on NSAIDs [3–7, 9, 11, 12]. This study, one of the few that has examined the use of self-medication in the South Asian region, looked at the use of NSAIDs, herbal/traditional medications and some selected medicines, and reports that self-medication is a frequent practice among local elite athletes. A direct comparison of self-medication prevalence rates in elite athletes in the developed world is not possible, since self-medication rates have not been included in most published reports [23]. In our study, self-medication was seen for both symptomatic pain relief and pain prophylaxis. Both allopathic and herbal/traditional medications were used by many athletes without a prescription or medical advice. Though a number of athletes used oral NSAIDs, the prevalence of use is much less than that is reported from studies in developed countries [23]. In a study that compared self-medication practices of world athletes in different continents, African and Asian track and field athletes reported using significantly fewer medications [5]. Cultural and economic factors may play a role here, and the reasons for the low prevalence would be worth investigating.

The marked differences in self-medication practices among different teams with kabadi and badminton having the highest prevalence and wrestling the lowest, may be possibly due to the differences in injuries and symptoms encountered in the different sports, views of the players, instructors and physiotherapists, even though these variables were not assessed.

Musculoskeletal pain followed by respiratory symptoms, fever and dysmenorrhea were common indications for self-medication, which corresponds with previous data from studies in the developed countries [6, 21]. Allopathic drug usage was most frequent for pain relief whereas herbal/traditional medications were used for respiratory symptoms which is a unique finding in this study. Pain prophylaxis was mainly practiced with non-prescription topical medicines which included topical NSAIDs and analgesic balms containing salicylates. The commonest medicine used for pain relief was paracetamol (acetaminophen) which is a non-prescription medicine in Sri Lanka, whereas among elite athletes in other studies, it was other NSAIDs, which falls into non-prescription category in the West [4–6, 12, 13]. Opioid analgesic use among the players was not reported in our study, whereas it has raised concerns in other parts of the world [24]. In Sri Lanka, all oral and topical NSAIDs are prescription medicines, except Aspirin. Though it has a relatively safer adverse effects profile than other NSAIDs, acetaminophen overuse may be associated with hepatic and renal dysfunction.

Despite regular use, close to 50% of athletes were unaware of possible adverse effects of self-medication.

Only 42.8% gave reasons for self-medication. More than 50% of participants not disclosing the reason and hence the unavailability of data on that aspect is a limitation of this study. The reasons for self-medication given are similar to those in a study among medical students in Nepal where mild illness, previous experience of treating a similar illness, and non-availability of health personnel were the most frequently cited reasons [17].

Athletes purchased medication by direct request, indicating the role of the pharmacist in facilitating undue self-medication practices. In Sri Lanka, the number of non- prescription drugs is very limited, but the general public frequently has access to prescription drugs without an authorized prescription due to violations of regulations by the National Medicinal Regulatory Authority [22]. Athletes appear to have rather unrestricted access to both non-prescription and prescription drugs, as well as herbal preparations, which are widely available in the market. This phenomenon is possibly compounded by the facts that the athletes themselves and their trainers not being fully aware about the potential harm caused by inappropriate practices of self-medication and the deficiencies of a proper documenting system to record the medication use by athletes at national level.

A significant finding is that family members rather than trainers or peers influenced practicing of self-medication, emphasizing the need to increase awareness of the dangers of self-medication through allopathic or herbal/traditional medications throughout the general population.

Reporting of adverse effects following NSAIDs in our study was lower than in the developed world [21], possibly due to lower frequency of use of oral NSAIDs. Use of antibiotics, particularly for respiratory tract problems and fever, was also noted, raising the possibility of unsupervised use of antibiotics causing microbial resistance.

Herbal/traditional medicines used by this population were mainly commercially prepared mixtures. Use of herbal/traditional medicine in Asian countries where such practices are prevalent among patients [19], has not been adequately studied so far among athletes. Herbals/traditional medicines also contain many substances/ingredients which have not been evaluated in the context of doping as well as their potential adverse effects and reactions with other concomitant medicines.

The response rates in most squads were excellent except for 4 squads, in which the response rates were approximately 40%. Cricket, rugby, swimming and basketball squads were unable to participate because of on-going tournaments, hence the actual figures on prevalence of self-medication in Sri Lankan athletes could be higher than those reported in this study.

Our study did not compare intake of medicines between athletes and non-athletes. There are reports of higher medicinal intake among athletes than non-athletes [23].

## Conclusions

Self-medication for symptom relief and prophylaxis was common among Sri Lankan national- level athletes and the patterns varied among different sport teams. Frequency of oral NSAID use is much lower than that is reported in non-Asian studies from developed countries. Both allopathic and herbal/traditional medication use were high, without adequate awareness about potential risks. Herbal medications were mainly taken as commercially available mixtures where the ingredients were often not listed. Prescription drugs such as oral NSAIDs and antibiotics were obtained without an authorized prescription. Pain prophylaxis was mainly achieved through non-prescription topical medications. Since attitudes towards self-medication practices were positive, increasing awareness is likely to have a positive impact on such practices. Future similar studies across the region are essential to assess the extent of the problem of self-medication and the prescribers’ role has to be emphasized regarding management of common problems of athletes.

**The questionnaire**      

## Abbreviations

NSAIDs: non steroidal anti-inflammatory drugs
WADA: World Anti-Doping Agency
LKR: Lankan Rupee
USD: US Dollar
